# Integration and Prototyping of a Pulsed RF Oscillator with an UWB Antenna for Low-Cost, Low-Power RTLS Applications

**DOI:** 10.3390/s21186060

**Published:** 2021-09-10

**Authors:** Stefano Bottigliero, Riccardo Maggiora

**Affiliations:** Department of Electronics and Telecommunications, Politecnico di Torino, 10129 Torino, Italy; riccardo.maggiora@polito.it

**Keywords:** elliptical dipole antenna, EM/circuit co-simulation, low-cost, low-power, power gating, RF oscillator, RTLS, ultrawide band antennas

## Abstract

The goal of this paper is to present a compact low-cost and low-power prototype of a pulsed Ultra Wide Band (UWB) oscillator and an UWB elliptical dipole antenna integrated on the same Radio Frequency (RF) Printed Circuit Board (PCB) and its digital control board for Real Time Locating System (RTLS) applications. The design is compatible with IEEE 802.15.4 high rate pulse repetition UWB standard being able to work between 6 GHz and 8.5 GHz with 500 MHz bandwidth and with a pulse duration of 2 ns. The UWB system has been designed using the CST Microwave Studio transient Electro-Magnetic (EM) circuit co-simulation method. This method integrates the functional circuit simulation together with the full wave (EM) simulation of the PCB’s 3D model allowing fast parameter tuning. The PCB has been manufactured and the entire system has been assembled and measured. Simulated and measured results are in excellent agreement with respect to the radiation performances as well as the power consumption. A compact, very low-power and low-cost system has been designed and validated.

## 1. Introduction

In recent years a great interest has been shown in UWB localization technology, demonstrated by the definition of the IEEE 802.15.4 standard for precision ranging [1]. The main reason is that its peculiar characteristics are suitable for high accuracy real time indoor localization [2]. In this paper we propose a compact, very low-power and low-cost solution for both the RF module of a transmitting tag that integrates a pulsed RF oscillator with a UWB antenna, and a digital pulse sequence generator that drives the RF oscillator circuit. The digital and RF modules are connected together to a rechargeable battery. The integration of the pulsed RF oscillator with the UWB antenna and the digital sequence generator is a major requirement in order to reduce the tag dimensions, manufacturing cost and power requirements. The main issue is to be able to tune the oscillator output with the antenna using the smallest PCB area possible. To achieve this result, it is important to understand the design implications when a real PCB is involved. Thanks to the EM/circuit co-simulation approach, we are able to evaluate the effects of the different PCB elements on the tag behavior.

Using a certain number of receiving sensors and a receiving computer it is possible to implement the localization engine for a multitude of such transmitting tags.

In the following Section 2 we will introduce the design details of both RF and digital modules. In Section 3 we will discuss the RF simulation setup and results. In Section 4 we will present measurement results of the manufactured prototype and in Section 5 we draw the conclusions.

## 2. Design

**Figure 1:**
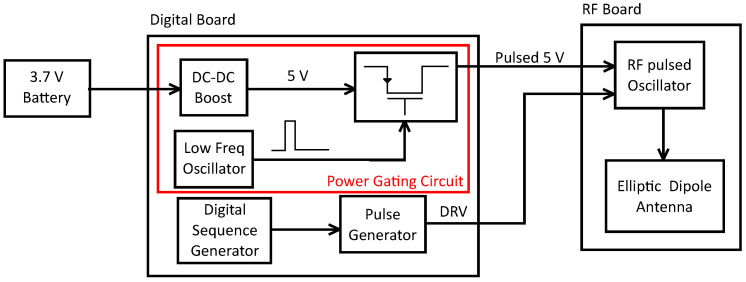
Tag’s high level block diagram. The digital board generates the pulsed voltage supply and the modulating pulse sequence for the RF oscillator.

The transmitting tag is composed of two boards. The first board hosts the digital and power gating circuit while the second board generates the carrier frequency using an RF pulsed oscillator driven by an external signal. The RF oscillator output is provided to the linear vertically polarized elliptic dipole antenna. The tag’s high level block diagram is shown in Figure 1 and the different blocks are described in the following.

**Figure 2:**
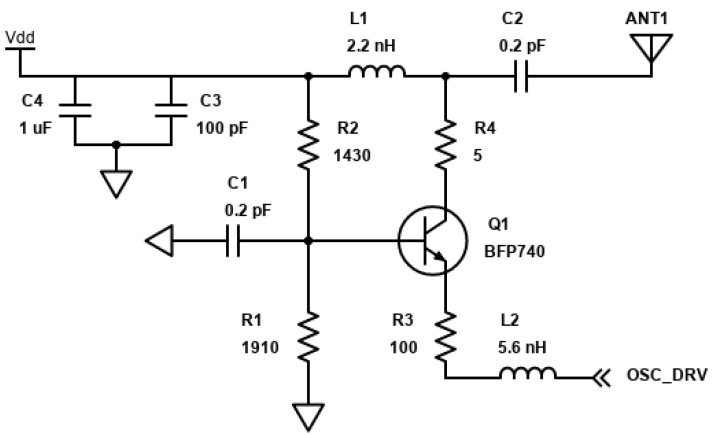
Schematic of the 7 GHz pulsed oscillator.

The RF pulsed oscillator circuit topology and its design methodology are presented in [3] where antenna and oscillator are on two separated printed boards. The design follows the method used for negative resistance oscillators where the Barkhausen criteria are satisfied so that the imaginary part of the input impedance, the one seen from the base of the transistor, is $Im(Z_{in})=0$ while the real part of the same impedance is $Re(Z_{in})<0$. To tune the resonance frequency is important to properly balance the reactance on the emitter to maximize the negative conductance at the base of the transistor. The oscillator operates in a common collector configuration; a command signal drives the emitter of the Infineon BFP740 SiGe-BJT transistor while the output is taken from the collector and sent to the antenna. The final RF pulsed oscillator schematic is shown in Figure 2.

The passive components values obtained during the design phase, are the starting point for the parameter tuning simulation phase to center the oscillating frequency at 7 GHz using a supply voltage of 5 V. Thanks to the co-simulation method, we are able to integrate the PCB, surface mount devices (SMDs) component and antenna contribution to the oscillator analysis, to estimate their effects, and to optimize the passive SMD components value accordingly. A RO4350B core 0.508 mm thick with very low losses (tanδ = 0.003) [4] has been adopted as dielectric substrate for the PCB hosting the RF pulsed oscillator and the antenna.

The results of the simulation phase are shown in Figure 2. In summary, to match the signal amplitude and frequency requirement, it was necessary to have a very small the capacitance at the base of the transistor and at the output of the circuit.

The antenna connected to the RF pulsed oscillator is a microstrip elliptic dipole antenna in linear vertical polarization. Microstrip antennas are inexpensive compared to ceramic chip ones currently on the market and have comparable performances [5]. An experimental study for UWB elliptic dipole antennas is presented in [6]. In this study only FR-4 and high dielectric constant materials were used.

In our case, we decided to adopt the same design methodologies but using different RF dielectric constant materials that allowed us to integrate the RF pulsed oscillator and the antenna on the same PCB and to reduce the overall dimensions. By changing the ratio between the minor semi-axis b and the major semi-axis a of the ellipses it is possible to extend the antenna bandwidth. Starting from the elliptic configuration, we optimized it. A unitary ratio between the semi-axis was sufficient to cover the required bandwidth of 500 MHz.

**Figure 3:**
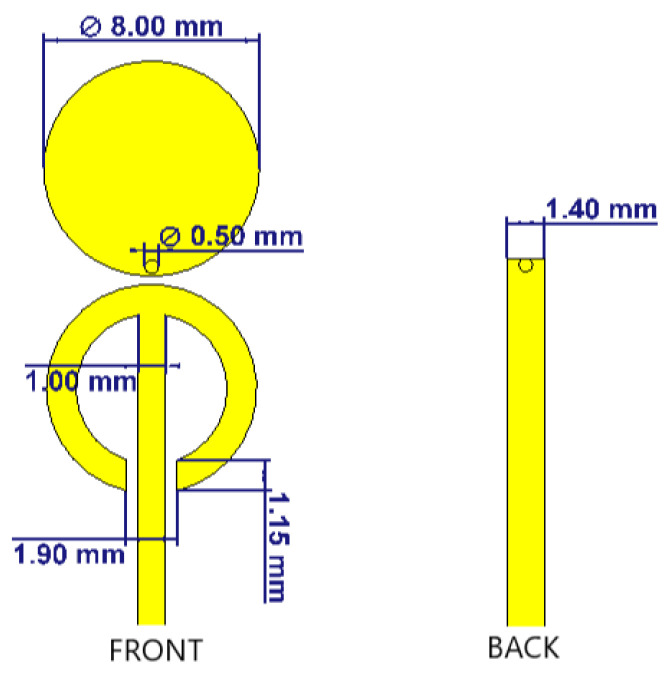
Antenna geometric parameters, front (**top**) view on the left and back (**bottom**) on the right.

The geometrical parameters of the optimized antenna are reported in Figure 3.

The digital sequence generator circuit provided the transmitted sequence signal to the RF pulse generator that implemented the 2 ns pulse signal driving the RF pulsed oscillator. The tag transmitted a sequence of pulses modulated using the On-Off Keying (OOK) technique. The whole sequence was hardwired for each tag and it was 15 bits long where the first 7 bits represented a preamble common to all tags and the latter 8 bits were a unique tag ID number. The preamble took the specific values of a Barker 7 code [7]. We used a modified version of the code where there were no pulses in correspondence of −1 in the sequence, this allowed for a simpler receiver architecture without losing the benefit of Barker codes. In this way, the carrier frequency generated by the RF oscillator was OOK modulated so that, when a sequence bit was equal to “1”, a pulse was transmitted, and when it was equal to “0”, no pulse was transmitted. This modulation simplified the tags’s hardware design dramatically, allowing the sequence generation circuit to be only the cascade of two 8 bit shift registers.

**Figure 4:**
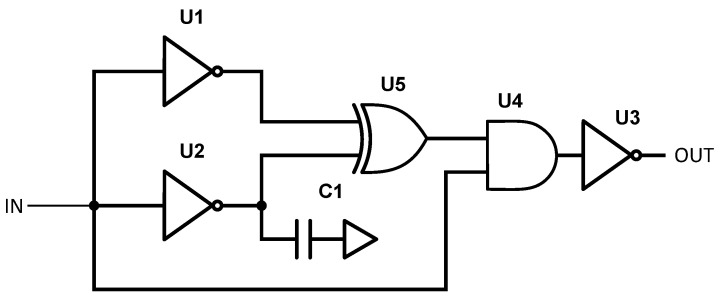
Low-cost 2 ns pulse generation circuit.

The separation in time between two subsequent pulses in a sequence was fixed by the 20 MHz (50 ns) clock signal used to time the shift registers. The sequence repetition frequency (SRF) was set by the power gating circuit (described in the following). The driving signal that modulated the carrier frequency was a 2 ns baseband pulse generated using the circuit shown in Figure 4. The implementation of short pulses may require very high speed and expensive hardware. Here, by using only discrete logic gates, we were able to maintain very low costs.

The transmitted sequence signal from the shift registers was provided at the input of two equal inverters where one of the two had an additional capacitive load at the output. This allowed us to increase the gate delay arbitrarily. A value of 33 pF for C1 was sufficient to add a delay of 2 ns. The pulse was obtained at the output of the XOR gate and its duration was proportional to the delay between the two inverters’ outputs. The pulse sequence went into an AND gate together with the original sequence signal in order to filter out a second unwanted pulse exiting the XOR gate. The signal was inverted and provided to the RF pulsed oscillator emitter.

**Figure 5:**
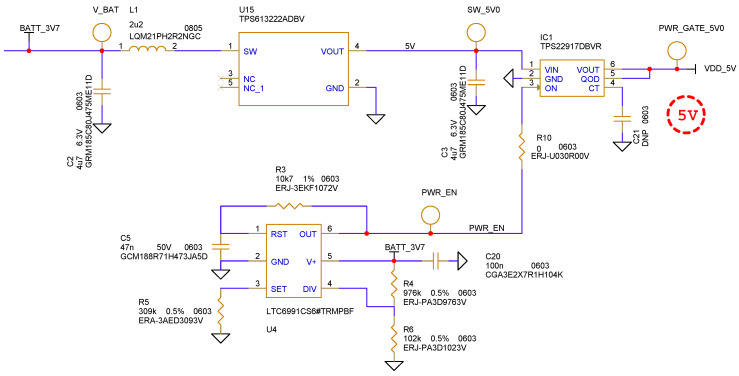
Power gating circuit implemented to reduce power consumptions. Only the LTC6991 low frequency oscillator and the DC-DC converter are always powered on.

The power gating circuit was the most efficient solution to drastically reduce the power absorption and its schematic is shown in Figure 5. The supply voltage of 3.7 V was obtained from a rechargeable Lipo battery. The battery voltage was up-converted using a switching DC-DC boost converter to 5 V. The higher supply voltage allowed us to obtain a higher RF output signal.

The output of the DC-DC converter was provided to a TPS22917 [8] switch from Texas Intruments. The On-Off state of the transistor was controlled by the LTC6991 [9] low frequency oscillator from Analog Devices. This component was the key element of the power gating circuit and allowed us to create low frequency, low duty cycle waveforms. Setting the value of R5 it was possible to set the SRF; in this case we set the value to have 20 sequences per second. The product of R3 and C5 set the duration of the pulse that drove the switch.

The entire transmission of a single 15 bit sequence lasted 750 ns, but in order to take into account the charge and discharge time of the power gating circuit, we had to set the driving pulse duration to a minimum value of 470 μs. In these conditions, both the RF oscillator and the digital circuit were power supplied for only 0.94% of the time for an SRF of 20 Hz (corresponding to a sequence repetition interval of 50 ms). If the application allowed, the SRF could be reduced to less than a repetition per second, further reducing the power consumption.

## 3. Simulation

The simulations of the antenna and RF pulsed oscillator assembly were performed using CST Microwave Studio adopting the EM/circuit co-simulation method [10]. Examples of usage of this method were presented in [11,12]: in both cases, the co-simulation method allowed them to integrate non linear component and SMD components in the 3D model. The EM simulation was set to have a port for each component in the PCB and to generate the complete scattering matrix of the 3D model and the farfield results.

**Figure 6:**
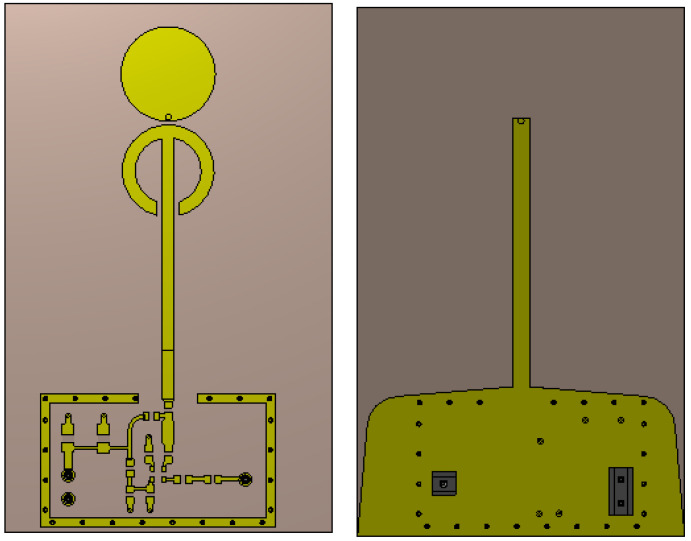
Top and bottom view of the PCB 3D model.

The 3D model of the PCB is shown in Figure 6.

**Figure 7:**
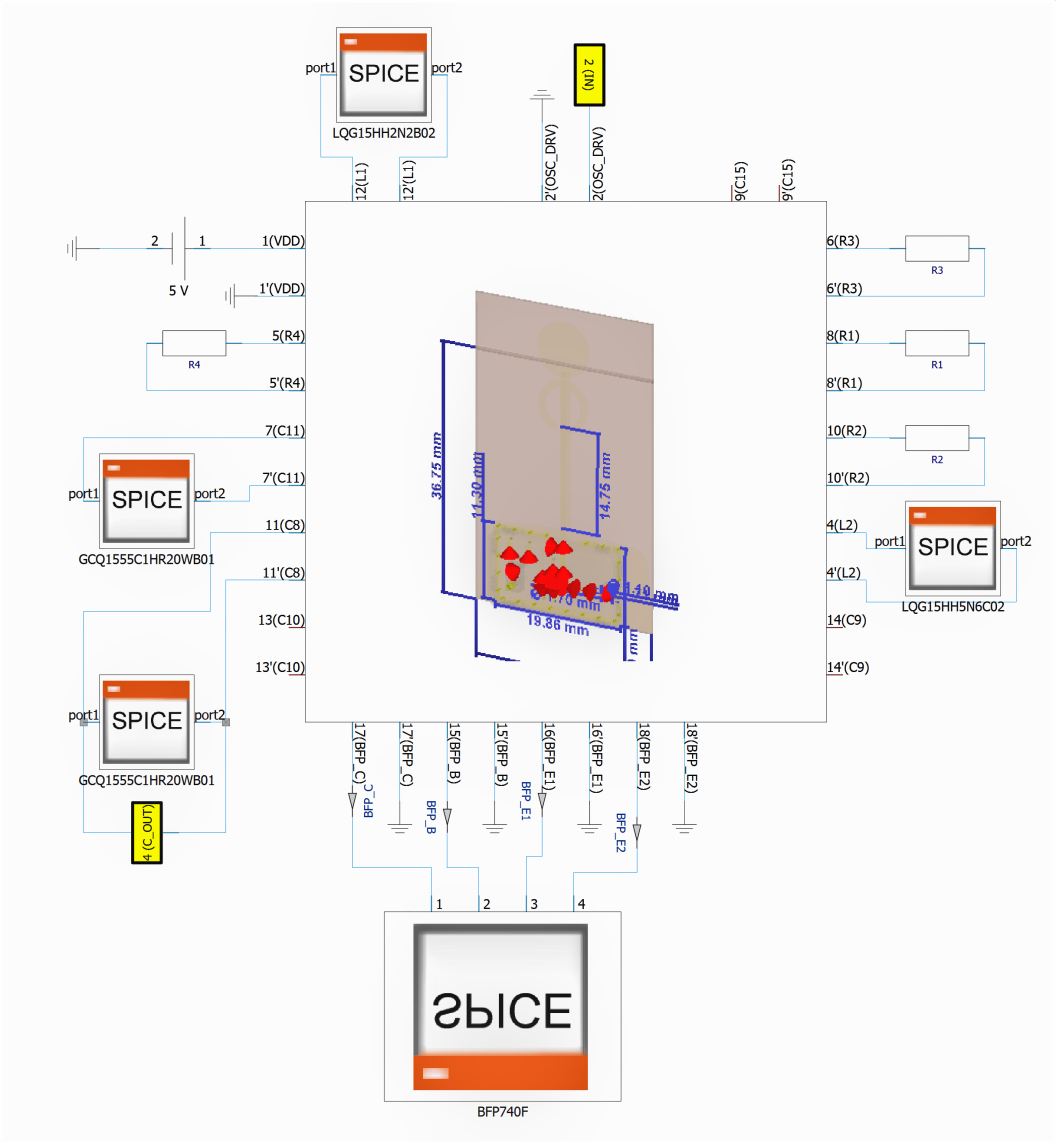
CST transient simulation schematic. The 3D model used in the EM simulation is instantiated as an N-port component.

The top view shows the dipole antenna, the oscillator circuit and a guard of ground vias while the bottom view shows the ground plane and the pin header used to power the board and to provide the driving signal to the RF pulsed oscillator. The EM simulation allowed us to have a full description of the PCB behavior and to see how it affected the tag functionality. For the complete tag simulation we connected the Gummen Pool SPICE model of the transistor [13] and of the SMD components to the PCB circuital n-port block and performed a transient simulation. In Figure 7 the complete schematic is shown. The part number and value of the simulated components are reported in Table 1.

The co-simulation allowed us to estimate the antenna radiation pattern and the shape of the output signal provided to the antenna. The radiation pattern simulated at 7 GHz is shown in Figure 10 with the blue dashed curve. The first two plots represent the $\phi =90^{\circ }$ and $\phi =0^{\circ }$ cuts while the third is the equatorial cut $\theta =0^{\circ }$ (as shown in Figure 9 where the z axis is parallel to the antenna polarization). The antenna main lobe was slightly tilted upwards and radiated almost uniformly in all ϕ directions.

**Figure 8:**
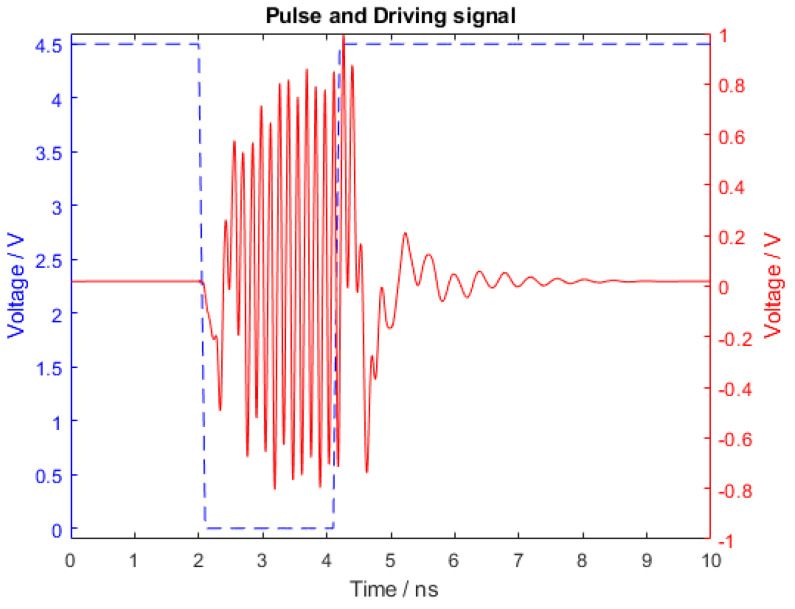
Voltage of the RF output across the output capacitor (red), and the driving signal (blue).

The transient simulations results are shown in Figure 8. The blue curve represents the command signal, simulated as 2 ns square pulse with 100 ps rise and fall times and 4.5 V amplitude; the red curve is the pulsed oscillator output. The signal had a peak to peak amplitude of 1.5 V and reached the 90% of the maximum amplitude in 2–3 carrier frequency periods. The circuit behaved as intended generating a 2 ns pulse at 7 GHz.

Furthermore, a functional simulation of the digital circuit was performed using PSpice to test the timing and feasibility of the power gating circuit.

## 4. Results

**Figure 9:**
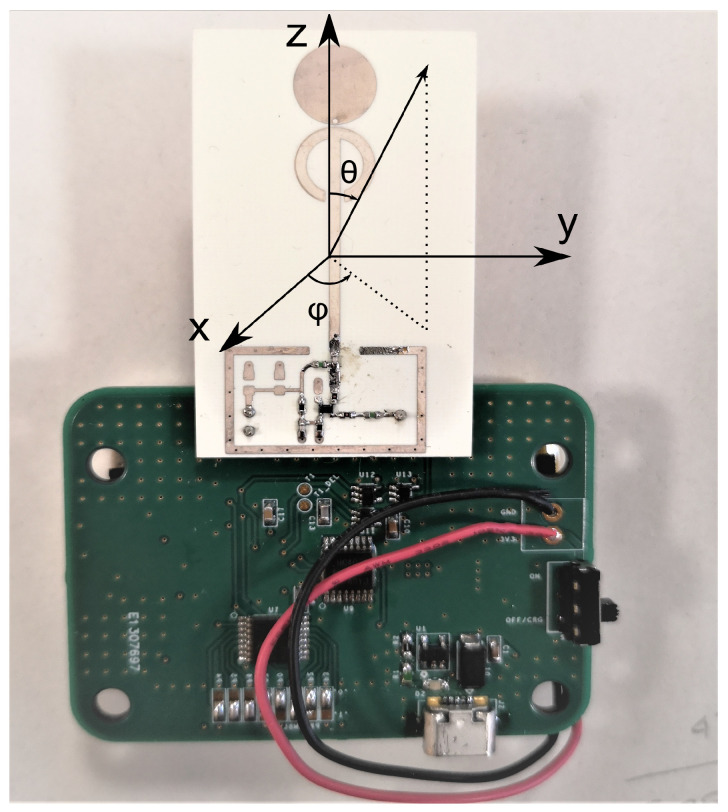
Assembled tag prototype with the oscillator and antenna board connected to the digital control circuit.

The tag was manufactured, assembled and measured. The final prototype is shown in Figure 9. It was possible to distinguish two different boards connected one on top of the other, the smaller one was the RF pulsed oscillator and antenna PCB while the other was the digital circuit board. The rechargeable battery was positioned on the bottom side of the digital circuit. The whole system dimensions were 75 mm × 55 mm × 10 mm making the whole tag smaller than a credit card. The tag did not require any programming since all parameters were hardwired.

To evaluate the radiation pattern, the tag was measured in the anechoic chamber of our institution. To perform this operation, the driving signal on the oscillator emitter circuit was fixed to ground setting the oscillator to work in a continuous wave (CW).

**Figure 10:**
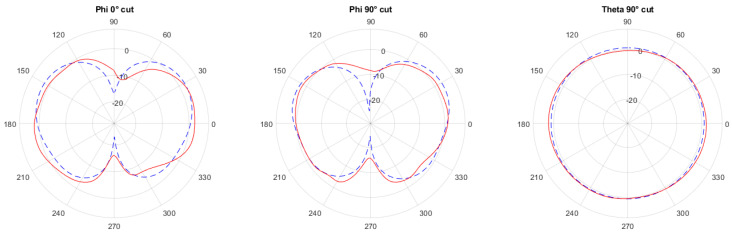
Comparison between the simulated radiation pattern (dashed line) and the measured one (red curve).

The measured radiation pattern is shown in Figure 10 with the red solid curve.

The results were post processed in Matlab using a cubic spline interpolation to reduce noise and normalized to the measured transmitted power of 7.5 dBm to properly compare to the simulated radiation patterns. The comparison between simulations and measurements showed an excellent agreement.

**Figure 11:**
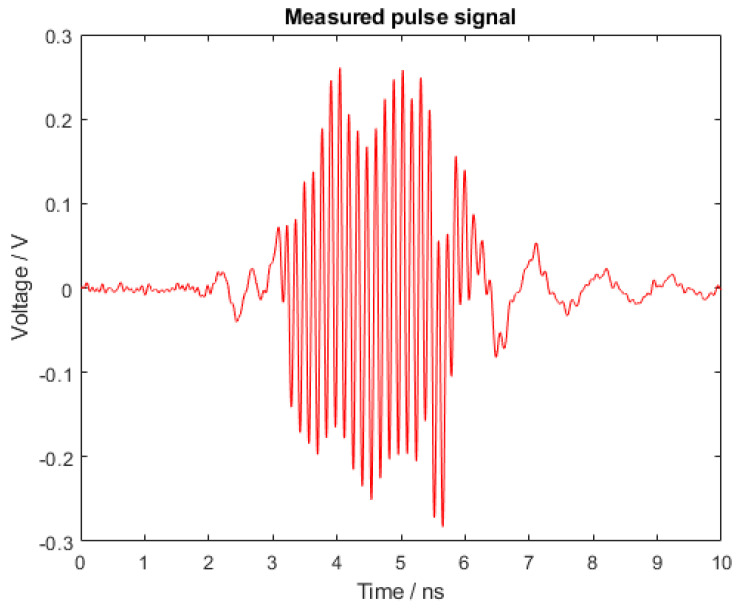
Voltage of the radiated pulse signal measured on the oscilloscope.

To test the pulsed behavior of the circuit, we connected the oscillator board to the digital control circuit. The signal radiated by the antenna was measured using a receiving antenna probe connected to an high frequency oscilloscope. The measurement setup was calibrated by comparing the output signal of an RF signal generator transmitting 0 dBm in two cases: (1) direct connection between the RF generator and the oscilloscope through a coaxial cable, and (2) an over the air configuration where the RF generator was connected to the tag antenna and spaced apart by a known distance equal to 1 mm from the probe antenna. The losses of the measurement setup were estimated to be equal to L = 9.5 dB. The tag signal measured with the receiving antenna probe at the same distance of 1 mm from the tag antenna is shown in Figure 11. The driving signal in this case was provided by the digital control circuit. The measured signal amplitude was comparable with the simulation results shown in Figure 8 once the calibrated losses, equal to 9.5 dB, were taken into account. The oscillation frequency was measured to be equal to 7 GHz.

Again, the comparison between simulation and measurement results showed an excellent agreement.

**Figure 12:**
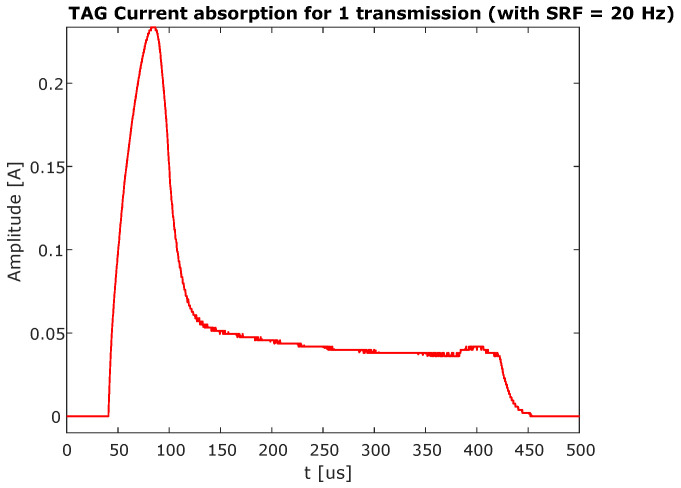
Measured waveform of the current absorbed from the battery, with focus on the 470 μs during the On state of the circuit. The waveform is obtained averaging 64 successive transmissions.

The tag power absorption and battery duration were estimated. The tag was connected to a laboratory power supply set at 3.7 V and to a high precision series resistor. By measuring the voltage across the resistor using the oscilloscope, and dividing it by the sensing resistor value, we obtained the current absorption for a single sequence transmission. In Figure 12 the detail of the current absorbed during the 470 μs interval during which the voltage supply was provided to the circuit is shown. We computed the average current consumption over the entire duration of the voltage supply pulse and obtained the average power of a single sequence transmission as

(1) $$P_{avg}=R\cdot I_{avg}^{2}=12.3\;\mathrm{mW}.$$

The energy consumed was equal to the average power of a single sequence transmission multiplied by the voltage supply pulse duration, in our case, 470 μs.

(2) $$E_{singleTX}=P_{avg}\cdot \tau =6\;\mu \mathrm{Ws}$$

In our case the SRF was set to 20 repetitions per second leading to a total absorption per second of $E_{abs}=120$ μWs.

The prototype used a 3.7 V Lipo battery with 1800 mAh current rating, for a total of 6.6 Wh. Taking the ratio between the battery energy rating and the absorbed energy per second we obtained a rough estimation of the battery duration of around 6 years. The energy absorbed by the system during the off time was negligible. These results can be further improved for those RTLS applications that allow less than 20 transmissions per second allowing the battery life span to increase further.

## 5. Conclusions

In this paper we proposed the design of a compact, low-cost, low-power UWB tag for RTLS application. The RF pulsed oscillator that generates the 7 GHz carrier signal is integrated on a single PCB integrating a custom UWB antenna. The EM/circuit co-simulation method allowed us to evaluate the effects of the PCB on the oscillation frequency and on the antenna radiation pattern and to tune the behavior of the oscillator using the component’s SPICE models. The designed solution is cost efficient both for components and bare boards and it is versatile in terms of channel frequency selection through components values tuning. The overall cost of the components and the PCBs is in the order of $10. The digital circuit designed to drive the oscillator is able to reduce the power absorption using power gating techniques drastically increasing the battery life.The power absorption of the entire system have been estimated by measuring the current absorbed from the battery over time and multiplying it by the battery voltage value. The tag has been manufactured to evaluate its performances in terms of radiation pattern, output signal amplitude and frequency and to compare them with simulated results.A comparison based on battery life, dimensions and cost between our tag and two high end industrial solutions [14,15] is reported in Table 2.

The obtained results shows that with the adopted method we have been able to integrate the RF oscillator and the UWB antenna on a single board and, together with the power gating technique and low-cost design choices, we obtained an UWB tag for RTLS applications performances better than some already on the market.

## Figures and Tables

**Table 1:** Details of the components used in the simulation.

Component	Value	Part Number	Manufacturer
C1, C2	0.2 pF	GCQ1555C1HR40BB01	Murata
C3	100 pF	GCG1885G1H101JA01D	Murata
C4	1 uF	GRT188C81A105KE13D	Murata
L1	2.2 nH	LQG15HH2N2B02D	Murata
L2	5.6 nH	LQG15HH5N6C02D	Murata
R1	1.91 kΩ	ERJ-2RKF1911X	Panasonic
R2	1.43 kΩ	ERJ-2RKF1431X	Panasonic
R3	100 Ω	ERJ-U02F1000X	Panasonic
R4	5 Ω	ERJ-U02F5R10X	Panasonic
Q1	BFP740	BFP740FH6327XTSA1	Infineon

**Table 2:** Comparison between this work and two main industrial solutions.

System	Battery Life (Years)	Dimensions (mm)	Tag Cost (USD)
Sewio	2.8	46 × 55 × 17	30–100
Simatic	1	62 × 95 × 13	30–100
This work	6	55 × 75 × 10	10–20

## Data Availability

Data can be provided upon request to stefano.bottigliero@polito.it.
